# Transparency ethics in practice: Revisiting financial conflicts of interest disclosure forms in clinical practice guidelines

**DOI:** 10.1371/journal.pone.0182856

**Published:** 2017-08-25

**Authors:** Yidan Lu, Derek J. Jones, Nour Sharara, Tonya Kaltenbach, Loren Laine, Kenneth McQuaid, Roy Soetikno, Venkataraman Subramanian, Alan Barkun

**Affiliations:** 1 Division of Gastroenterology, McGill University Health Center, Montreal, Quebec, Canada; 2 Faculty of Law, McGill University, Montreal, Quebec, Canada; 3 Hospinnomics, Paris School of Economics & Public Hospitals of Paris, Paris, France; 4 Division of Gastroenterology, San Francisco Veterans Affairs Medical Center, San Francisco, California, Unites States of America; 5 Division of Gastroenterology, University of California, San Francisco, California, Unites States of America; 6 Section of Digestive Diseases, Yale School of Medicine, New Haven, Connecticut, Unites States of America; 7 Division of Gastroenterology, Veterans Affairs Connecticut Healthcare System, West Haven, Connecticut, Unites States of America; 8 Division of Gastroenterology, Veterans Affairs Palo Alto Healthcare System, Palo Alto, California, Unites States of America; 9 Division of Gastroenterology, Stanford University School of Medicine, Palo Alto, California, Unites States of America; 10 Division of Gastroenterology, University of Leeds, Leeds, United Kingdom; 11 Department of Epidemiology, Biostatistics and Occupational Health, McGill University, Montreal, Quebec, Canada; York University, CANADA

## Abstract

**Background:**

Authors of clinical practice guidelines (CPGs) disclose financial conflicts of interest (FCOIs) to promote transparency ethics. Typically, they do so on standard declaration forms containing generic open-ended questions on FCOIs. Yet, the literature is scant on the format and effect of alternative disclosure forms. Does supplementing a standard form with subsequent detailed disclosure forms tailored to the context of the CPG improve the yield or accuracy of FCOIs declarations?

**Methods:**

For an international CPG in gastroenterology on the endoscopic surveillance for colorectal neoplasia in inflammatory bowel disease, we compared the use of a standard FCOIs disclosure form with a contextual FCOIs disclosure form that detailed commercial relations related to the CPG topic. This included manufacturers of endoscopes, endoscopy equipment and accessories. Participants completed the generic form early, and the supplementary contextual form six months later. We then compared the FCOI disclosures obtained.

**Findings:**

26 participants provided FCOIs disclosures using both disclosure forms. We found discrepancies regarding (1) the disclosure of FCOIs (presence/absence), and (2) the listing of financial entities. While the number of participants who disclosed a FCOI remained the same (30.8%) using the two forms, disclosures were not from the same individuals: two additional participants disclosed a FCOI, whereas two participants withdrew previous disclosures. Among those who reported a FCOI in either form, we noted inconsistencies in disclosures for 70% of the participants. This included changes in FCOIs disclosure status or modifications of "their commercial relations".

**Discussion:**

Accurate reporting of FCOIs advances the transparency and ethical integrity of CPGs. Our experience suggests that a contextual FCOIs disclosure form tailored to content of the CPG with narrow, detailed questions provides supplementary, more complete FCOIs declarations than generic forms alone. The finding raises challenges on how forms are best written and formatted, optimally timed, and more effectively processed with sensitivity to professional behaviour, so as to heighten transparency.

## Introduction

Clinical practice guidelines (CPGs) synthesize and appraise the available scientific literature to produce recommendations that assist health care decision-making. [1] They are defined by the Institute of Medicine (IOM) as “statements that include recommendations intended to optimize patient care that are informed by a systematic review of the evidence and an assessment of the benefits and harms of alternative care options”. [1] CPG standards and methodologies have significantly evolved since the 1990s, [2] along with an exponentially expanding number of CPGs being produced. The Guideline International Network currently counts over 6300 guidelines from 85 countries, [3] almost doubling since 2011, when it documented over 3700 guidelines from 39 countries. [1] Despite such proliferation, many have disputed the accuracy and integrity of certain CPGs [4] and warned about the limitations, and potential harms from guidelines that fail to meet quality standards. [5]

Even in an era of evidence-based medicine with highly structured approaches to grading the quality of the evidence and strength of each recommendation, CPG developers still confront incomplete, imperfect or contradictory scientific evidence. Data and evidentiary voids on CPGs issues sometimes oblige participating experts to make subjective assessments. Recommendations are therefore influenced by the guideline panelists’ personal input and perceptions, especially when the science is unclear. In situations where the quality of CPGs is compromised, the value of the recommendations produced is undermined. The impact may reach medical standards, policy and financing, but “[t]he greatest danger of flawed clinical guidelines is to patients”, potentially resulting in “suboptimal, ineffective, or harmful practices.” [5] Indeed, “flawed” CPGs can, for instance, provide inaccurate or biased clinical information, impose unnecessary interventions, or have limited applicability.

Unsurprisingly, then, to ensure the quality, impartiality and ethical integrity of guidelines, conflicts of interest (COIs) in CPGs have been the subject of increasing awareness, scrutiny and standards. [6, 7] The CPG process has been critiqued for pervasive and predominant, undisclosed, underreported and inaccurate, or poorly managed financial COIs (FCOIs). [8–12] Accordingly, an important focus over the last decade has been on understanding, identifying and addressing FCOIs as a source of bias in CPGs. In various countries, the management of FCOIs has been recommended or mandated. [1, 13] A 2011 report from the IOM addresses the challenges posed, and notes the role of transparency in the CPG process. Drawing on interdisciplinary literature, the IOM urges “provision of information to CPG users that enables them to understand how recommendations were derived and who developed them”. [1] Transparency, it is thought, promotes accountability to professional peers by divulging potentially biasing factors. Such transparency, in turn, is thought to advance impartial, independent CPGs. Doing so is consistent with ethical values of candor, virtue and trustworthiness.

In practice, the disclosure of FCOIs is the enabling process for transparency. Indeed, disclosure has become the implementing transparency norm over the last decade. [1, 6, 14] The literature indicates that even if a lack of uniform international standards raises important ethics challenges for transnational CPGs, CPGs grounded on impartiality depend on effective process and forms for FCOI declarations, as integral parts of coherent CPG ethics frameworks. [15] Typically, FCOIs declarations are reported on generic forms with open-ended questions. [16] They are completed early in the CPG process, and generally consist of standard forms issued by the guideline developing body, and are thus not tailored to the topic of the CPGs. The forms typically require CPG participants to list any relevant financial COI, and may include additional questions detailing the COIs such as the identification of commercial interests involved, the nature of the financial relationship, and the amounts received. The management of COI is contingent on the proper disclosure and documentation of COIs. However, there is a paucity of data on the structure of COI disclosure forms that optimally captures relevant FCOI disclosures with high accuracy.

In this research brief, we therefore compare the results of a novel supplementary contextual disclosure form with a standard form alone for an international CPG. [17] A "contextual" disclosure form is specifically tailored to the CPG topic, to enable the declaration process by identifying commercial entities and relationships that may present a FCOI. It is completed later in the CPG process, to supplement standard original FCOIs declaration forms. We assessed declarations from both forms, to determine whether a supplementary FCOIs declaration document yields additional FCOI information.

## Methods

As part of the development of an international CPG on the surveillance for colorectal neoplasia in inflammatory bowel disease, we compared the results of FCOI declarations from two different FCOIs disclosure forms completed by CPG participants. [17] Each participant first completed a standard FCOIs disclosure form issued by the guideline’s organizing scientific body. Six month later, at the consensus meeting, they completed a supplementary, detailed and contextual FCOIs disclosure form specifically tailored to the CPG. An *ad hoc* ethics committee was created to oversee COI issues and this study on COI disclosures.

The international CPG was developed by panel 21 international experts from North America, Europe and Asia (physicians, methodologists, a nurse, and a patient representative), as well as 8 non-voting participants included for content expertise. [17] All participants who were neither members of the ethics committee nor members of the CPG’s steering committee were invited to participate in this COI study. The scope of participation was designed to maximize efficacy of the two different disclosure forms, and to preserve the accuracy of disclosures.

In the absence of uniform international standards, we adopted institutional COI norms of one of the key scientific societies for the consensus meeting, the Canadian Association of Gastroenterology (CAG). CAG has prior experience in CPG development. [18, 19] Six months prior to the CPG consensus meeting, participants completed CAG’s mandatory, standard FCOI disclosure form. (S1 File) The form features a blank table for participants to disclose relevant financial relations, between participants or family members and commercial entities, over the last 24 months. The form defines FCOIs as “when outside financial interests or connections influence the ability to act with integrity, objectivity and independence with regards to the assigned task, and encompass actual, apparent or potential COIs." The definition derives from CAG adoption, with some of modification [20, 21] of first generational definitions of FCOIs, such as those of the US Accreditation Council for Continuing Medical Education. [22, 23] Such first generation definitions have since evolved with expanded concepts of COI by public analysts like the IOM. [24] These second generation approaches often broaden COI definitions, sometimes with precise elements like primary and secondary interests, monetary thresholds and varied time periods for disclosure. [13, 25] Those precisions influenced our next steps.

To provide context and details that might facilitate the disclosure process, the *ad hoc* ethics committee developed a supplementary contextual FCOIs disclosure form. (S2 File) The supplementary form specifically addressed issues, financial relations and commercial entities identified by the ethics committee as pertinent and posing a potential risk of COI to the specific CPG topic and recommendations. Since part of the CPG addresses chromoendoscopy—a technique that uses dyes to identify colonic neoplasia during endoscopy—relevant manufacturers of dyes, endoscopes, and endoscopy equipment (e.g., spray catheters, injection needles) were thus specified on the supplementary form. The form also detailed a checklist of prompters on the nature and extent of the financial relation: e.g., range of monetary amounts, and roles played. (Table 1)

**Table 1:** Elements of a contextual financial conflicts of interest disclosure form.

**Timing of disclosure**
Declarations of FCOIs repeated at a time close to the final voting or final consensus meeting
**List of all relevant companies related to the topic of the clinical practice guideline**
Direct and indirect associations When possible, include all pertinent commercial entities that extend to different countries and professional background to reflect the composition of the participants
**Checkbox for details of each disclosed FCOI**
Role Amount Nature Timing (past and future)

FCOI = financial conflict of interest

The supplementary form was completed during the last stages of the guideline development, when participants assembled at the CPG consensus meeting for the final discussion and voting on recommendations. In an ethics process session at the meeting, prior to final voting, participants were invited to complete this supplementary contextual FCOIs disclosure form. (S2 File) Participants also then answered a research questionnaire on COI perception and management, at the ethics session. (S3 File) Institutional research ethics approval was obtained from McGill University Health Center, and participants provided written informed consent.

### Role of funding source

The CPG process received funding from the Maxine and Jack Zarrow Family Foundation and Warren K Warren Foundation, which otherwise had no involvement in this study. [17]

## Results

26 out of 27 participants completed the two FCOIs disclosure forms and answered the questionnaire on their perception of FCOIs in the guidelines process. The latter revealed that all participants (26/26) believed that FCOIs need to be disclosed. However, many of them (46%, 12/26) deemed that FCOIs disclosure alone is insufficient to allow full participation in the CPG process, agreeing that additional FCOIs management strategies may be necessary for participants with FCOIs.

In the context of such perceptions on operationalizing transparency, we compared participants' declarations of FCOIs through the standard disclosure form with those from the contextual disclosure form. We found discrepancies in the declared content, and presence/absence of FCOIs. While the number of participants disclosing at least one FCOI remained the same (30.8%, 8/26), the disclosures were not from the same individuals: two additional participants disclosed a FCOI; two participants with previous declarations withdrew them. When responses from the two forms were combined, the number of participants to declare at least one FCOI increased from 30.8% to 38.5% (10/26). Inconsistencies in reporting were noted in 7/10 –(70%; 95% CI: 41.6–98.4%) of the FCOI declarations. The discrepancies encompass changes in FCOIs disclosure status and modifications in the content of disclosed commercial relationships and interests (Table 2). For instance, one participant who disclosed COIs in both forms listed different commercial interests on each form (Table 2, participant ID 4). Another disclosed several COIs in the standard form, but indicated “no COI to disclose” in the second form (Table 2, participant ID 7). A list of modifications is included in the footnotes of Table 2.

**Table 2:** Summary of financial conflict of interest disclosure using a standard disclosure from and contextual disclosure form.

Participant ID	Standard FCOI disclosure form	Contextual FCOI disclosure form	Change in disclosure status	Change in disclosure content only	Description of change
1	Y	Y	N	N	No change
2	Y	Y	N	N	No change
3	Y	Y	N	N	No change
4	Y	Y	N	Y	Change in disclosure content ^1^
5	Y	Y	N	Y	Change in disclosure (new FCOI added) ^2^
6	Y	Y	N	Y	Change in disclosure content^3^
7	Y	N	Y	-	Disclosure in standard form but not in contextual form ^4^
8	N	Y	Y	-	No disclosure in standard from but disclosure in contextual form ^5^
9	Y	N	Y	-	Disclosure in standard form but not in contextual form ^6^
10	N	Y	Y	-	Declared "no" in both forms, but still disclosed and listed commercial entities in contextual form ^7^
**Total (Y)**	**8**	**8**	**4**	**3**	**Total with changes 7**

COI = conflict of interest, FCOI = financial conflicts of interest, ID = identification, N = no, Y = yes.

^1^ This participant disclosed four COIs with commercial entities in the standard form. In the contextual form, the participant disclosed a COI with only one commercial entity (different from the prior four disclosed). None of the four COIs initially disclosed (standard form) were part of the listing of relevant commercial interests, however the one COI disclosed using the contextual form was with a commercial entity included in the listing.

^2^ This participant disclosed two COI in the standard form. With the contextual form, the participant again disclosed the same two COIs, and added two additional commercial entities that the ethics committee had identified as relevant commercial entities (for a total of four COIs).

^3^ This participant disclosed financial relations with 50 commercial entities in the standard disclosure form. Only one of the entities had been identified as a relevant commercial entities provided in the contextual form. In the contextual form, the participant answered “yes” to having COIs, but did not name or detail them.

^4^ This participant disclosed financials relations with seven commercial entities in the standard disclosure form. None of the seven had been identified as relevant commercial entities listed in the contextual form. In the contextual form, the participant disclosed no COIs.

^5^ This participant answered “no COI to disclose” in the standard form. However the participant disclosed three COIs with commercial entities listed in the contextual form.

^6^ This participant disclosed COIs with 17 commercial entities in the standard disclosure form. Only one of them was included in the listing of relevant commercial entities provided with the contextual form. In the contextual form, the participant did not disclose any COI.

^7^ This participant answered “no COI to disclose” in both the standard and contextual form. However, in the contextual form the participant identified a COI with one commercial entity identified as relevant commercial entities detailed in the contextual form.

## Discussion

Our exercise documented important congruities and divergences in the evolving state-of-the art of identifying and managing FCOIs in the CPG process.

First, for instance, in response to our COI questionnaire, participants unanimously agreed that authors of CPGs must disclose COIs. If it has taken some two decades [26] for disclosure to become an established norm, it functions today as a basic, if imperfect standard and tool of COI ethics for CPGs. Disclosure remains an imperfect tool, in practice, as understanding of its actual working have evolved to the point that even the literature that acknowledges its role and beneficial effects also indicates "disclosures is no panacea," and may generate counter-intuitive, adverse effects. [27] For instance, studies have shown that through a behavioral phenomenon of "moral licensing," disclosure sometimes frees conflicted professionals to give more biased advice. [28] Further understanding of such phenomena should help improve and reform disclosure in practice.

Second, our participants divided over how best to manage FCOIs in the CPG process beyond basic disclosure: 55% thought disclosure suffices; 45% thought disclosure insufficient. The disagreement reflects divergence from, and continuing debate over, an emergent norm that has come into prominence in FCOI management within the last decade. Proponents of the norm reject sole reliance on disclosure as a minimalist management strategy that ignores refined principles of contemporary FCOI ethics, such as the principle of "proportionality." It holds that CPG participatory standards should be commensurate with COI risks. [15] Proportionality thus relies on a range of FCOI management tools to addresses COIs risks that range from minimal, to moderate, to high. On this logic, the IOM, the academic literature, institutions and increasing COI ethics policies now invoke FCOI management tools beyond disclosure for significantly conflicted experts. [6] Disclosure begins the FCOI management process. For moderate to high COI risks, it may extend to limiting conflicted experts participation in, or outright exclusion—with narrow exceptions—from CPG leadership roles, drafting, discussing, voting, etc. Our participants' difference of views on this strategy captures transitional dynamics of this emergent ethics norm.

Such emergent and pluralistic ethics norms—whether minimalist, proportionate or more robust—build on disclosure. For example, a strict avoidance strategy for FCOIs looks beyond managing them to avoiding them. Strict avoidance includes in CPGs only experts without FCOIs. Doing so maximizes impartiality and works well on issues where expertise without commercial relations is readily identifiable. Yet, the strategy risks silencing or compromising essential expertise in areas where the experts are few and the FCOIs minimal or manageable. [15] Accordingly, more permissive strategies grant experts with FCOIs who offer essential expertise a limited participatory role such as participating in the discussion process, but not in voting on recommendations [1]. Such management or avoidance of FCOI begins with identifying participants’ FCOIs. Such identification depends in turn on accurate, effective disclosures.

Thirdly, our study thus documents divergence between the principle of transparency and the actual practice of declaring financial interests with disclosure forms. Declarations of FCOIs function at the crossroads of disclosure ethics for CPGs. Declarations depend on honest self-reporting, framed by FCOIs procedures and disclosure forms. Disclosure forms translate the ethical principle of transparency into practice; by applying COIs definitions, monetary disclosure thresholds, the scope of financial relations, etc., to the declaration process. Their effectiveness depends on diverse factors: their interpretation, clarity and relevance, length or brevity, etc. The forms must strike balances. Given such considerations, a deeper understanding of their effectiveness is compromised by scant literature on their comparative design, impact and actual workings. The literature holds few, if any, empirical studies of the effectiveness of alternative FCOIs disclosure forms for CPGs.

In our study of disclosure forms and process, we evaluated the yield of administering two FCOI disclosure forms at two different times. We supplemented the original generic form with a detailed contextual form administered six months later. The latter afforded participants a second opportunity to refresh their recollection and to declare financial interests with more precise information, checklists and questions on relevant commercial relations. The result of administering both yielded more and revised FCOIs declarations. When we compared FCOIs declared on the contextual and generic forms, among those who reported a FCOI in either form, we found discrepancies in 70% of them (Table 2).

Our findings demonstrate important variability in disclosed FCOIs between a standard generic and a supplementary contextual disclosure form. How so? We discern an interplay of temporal, content, interpretive, and behavioral factors. If some revisions came from the later administration of the contextual form, timing alone unlikely accounts for their magnitude. Facial accountability to peers, [26] and the immediacy and synergy of the CPG meeting may have also prompted participants towards more probing analysis of their financial relations. They did so with more enabling disclosure forms that detailed a checklist of relevant commercial entities and products known in the clinical field. Such specificity likely functions as contextual enabler to the declaration process to heighten the utility of the supplementary form. Indeed, detailed and precise content in FCOIs declaration forms reduces ambiguity and generality, [29, 30] thus diminishing the risks of forgetting, ignoring, misinterpreting or misreporting relevant, significant financial relations. As such, the effectiveness of a FCOI disclosure form may be enhanced or compromised by the degree of clarity, context and precision on key conceptual and operational issues. For instance, if a form requires disclosure of "direct" and "indirect" or "significant" financial relations with "relevant" commercial entities but does not meaningfully define or illustrate the concepts, what shall participants declare? Some may judge as insignificant, and not declarable, $5000.00 in research support from a manufacturer of a device moderately implicated by a CPG. Other participants may reach the opposite conclusion. Greater clarity, precision, and contextual guidance likely reduce the risk of erroneous (non) declarations. We judge participants’ revised declarations as likely more accurate, because they flow from non-generic, narrower questions and contextual FCOIs information.

Our findings mirror similar results from studies on disclosure forms for authors of medical articles. Generic FCOIs disclosure forms with opened-ended questions prove sub-optimal due to their generality and vagueness; forms with narrow, contextual, and detailed prompters prove more effective partly because precision leaves less room for interpretation. [29, 30] If such findings suggest a need to revise generic open-ended forms, the insights remain indicia that merit further rigorous interdisciplinary reflection and research. Some research indicates, for instance, that whether, when and where one signs and pledges honesty in formal declarations influences ethical accountability and accuracy of completed forms. [31] Studies on "framing effects" further suggest that how a question or issue is framed may generate automated versus thoughtful ethical responses. [32] Finally from a behavioral science perspective on "nudge-theory" ethics, the second contextual form arguably functioned as a more potent moral symbol of ethical conduct, which effectively nudged professionals towards higher ethical vigilance, accountability [33] and enhanced disclosures once peers were assembled.

## Limitations

The small number of participants limits the generalizability of findings. A lack of uniform, harmonized international FCOI standards likely makes results contextual. The use of more or less stringent FCOI definitions, thresholds, disclosure and participation criteria may influence results. Our study design did not control for behavioral, interpretive, temporal or form-content dimensions of FCOIs declarations. Larger studies in future research may do so, to build empirical evidence of effective FCOIs disclosure forms and practice.

## Conclusions

The declaration of FCOIs remains a critical component of the ethical integrity of CPGs. Full, accurate disclosures advance transparency ethics. Un-declared, inaccurate or under-declared FCOIs undermine transparency. Our experience with generic and subsequent contextual FCOIs declaration forms in an international CPG yielded higher disclosure rates. The results suggest that precise, contextual content of disclosure forms may effectively interface with temporal, interpretive and behavioral factors to enable more complete, accurate declarations. The search for optimal FCOIs forms and disclosure process should continue with heightened analyses and research on how such factors best interface to advance transparency ethics.

## Supporting information

S1 File Standard financial conflicts of interest disclosure form. (PDF)

S2 File Supplementary contextual financial conflicts of interest disclosure form. (PDF)

S3 File Questionnaire on the perception of financial conflicts of interest. (PDF)

S4 File Financial conflict of interest disclosure of the SCENIC guideline panel members. (DOCX)
