# Supplementary material for: Transparency ethics in practice: Revisiting financial conflicts of interest disclosure forms in clinical practice guidelines
Source: PLoS One. 2017 Aug 25;12(8):e0182856. doi: 10.1371/journal.pone.0182856 (PMC5571907; doi:10.1371/journal.pone.0182856)
Supplement: S1 File — (PDF) [file pone.0182856.s001.pdf]

FINANCIAL INTEREST DISCLOSURE FORM

Objective:
The purpose of this guideline is to provide guidance about disclosure. Use it to assist in insuring you are not in actual, apparent or potential conflict of interest over the past 24 months.

Definitions:

1. An actual conflict of interest occurs when outside interests or connections (business, personal, professional, volunteer or other) influence your ability to act with integrity, objectivity, and independence in regards to the assigned task.
2. An apparent conflict of interest occurs when outside interests or connections (business, personal, professional, volunteer or other) appear to influence your ability to act with integrity, objectivity, and independence towards the assigned task.
3. A potential conflict of interest arises when you become aware of outside interests or connections (business, personal, professional, volunteer or other) that if acted upon may influence your ability to act with integrity, objectivity, and independence in completing an assigned task.

Determination of Conflict of Interest:
In order to determine if any type of a conflict listed above is possible with your assigned task, ask yourself the following question.

"Would my peers or the general public consider my presentation (judgment) to be biased or would they trust my decision if they knew of this particular situation?"

Example 1) You are asked to give a talk on treatment of a specific disease and you sit on a pharmaceutical advisory board of a drug that is used to treat this disease.

Example 2) You are chairing a committee charged with developing guidelines for the use of product X. You serve as a consultant for the company that makes product X.

Disclosure:
You must take reasonable steps to disclose circumstances that occurred over the past 24 months that may place you in actual, apparent or potential conflict of interest. When you identify that conflict has arisen or has the potential to arise, disclosure is required. Furthermore, you must disclose what steps will be taken to protect objectivity and to deter potential criticisms that might arise from actual, apparent or potential conflict of interest.

In regards to Example 1) above: you offer to speak on the pathophysiology rather than the treatment of disease, or you offer to have your talk about treatment vetted by the conference session organizer prior to the presentation at the meeting; In regards to Example 2) above: as a guideline organizer, you arrange to have another committee member 1) chair the session on treatment and 2) have final approval of the guidelines on treatment.

If in doubt about actual, apparent or potential conflict of interest, ask your committee chair (session organizer) for an opinion.

FINANCIAL INTEREST DISCLOSURE FORM

Activity:

The SCENIC Recommendations: Surveillance for Colorectal Endoscopic Neoplasia detection and management in Inflammatory bowel disease patients – international Consensus

Name: ___________________________________

We must ensure balance, independence, objectivity, and scientific rigour in all business, educational and scientific activities. Accordingly, you are expected to disclose any potential, apparent or actual interests or connections (business, personal, professional, volunteer or other) appear to influence your ability to act with integrity, objectivity, and independence towards the assigned task. Furthermore you must disclose what steps will be taken to protect objectivity and to deter potential criticisms that might arise from actual, apparent or potential conflict of interest. The intent of this initiative is to provide interested parties with information on the interests or relationships that could influence interpretations, recommendations, and conclusions.

Over the past 24 months, have you or your spouse received financial support or had a financial relationship from a commercial entity for any activity which may be perceived as being connected with this initiative?

☐ **NO**, I do not have any relevant financial relationships with any commercial interests. I agree to immediately notify the CAG National Office if a significant financial interest develops at any time prior to or during my participation in the above educational event, presentation or task

☐ **YES** If yes, please complete the attached table listing:

1) The company(ies), what was received, for what role? For example: speaker's bureau, consultant, research support, company advisory board, employee, stockholder, other. Please include additional pages, as required.
2) Describe steps you would take to resolve any potential, apparent or actual conflict of interest. For example: excuse yourself from voting on decisions involving the commercial interest, or talk vetted by individual with no conflict of interest.

Signature_______________________________________Date____________________________________

References:

1. Accreditation Council for Continuing Medical Education (ACCME). ACCME Standards for Commercial Support: Standards to Ensure the Independence of CME Activities. 2004. Available from: http://www.accme.org/index.cfm/fa/Policy.policy/Policy_id/9456ae6f-61b5-4e80-a330-7d85d5e68421.cfm
2. Wazana A. Physicians and the pharmaceutical industry. Is a gift ever just a gift? JAMA 2000;283:373-380.
3. Tenery Jr. RM. Interactions between physicians and the health care technology industry. JAMA 2000;283:391-393 .
4. Martin JB, Kasper DL. In whose best interest? Breaching the academic - industrial wall. NEJM 2000;343:1646-1649.

FINANCIAL INTEREST DISCLOSURE FORM

Commercial Interest	Nature and resolution of relevant financial relationship		
	What was received?	For what role?	Planned resolution
Example: Company X	*Honorarium*	*Speaker*	*Talk vetted by session leader*
