# Supplementary material for: Transparency ethics in practice: Revisiting financial conflicts of interest disclosure forms in clinical practice guidelines
Source: PLoS One. 2017 Aug 25;12(8):e0182856. doi: 10.1371/journal.pone.0182856 (PMC5571907; doi:10.1371/journal.pone.0182856)
Supplement: S2 File — (PDF) [file pone.0182856.s002.pdf]

FINANCIAL CONFLICT OF INTEREST DISCLOSURE FORM
SCENIC CONSENSUS MEETING 2014

Name: ______________________________

Objective

The purpose of this guideline is to provide guidance about financial conflict of interest disclosure. Use it to assist in identifying and disclosing actual, apparent or potential financial conflict of interests over the past 24 months.

Definitions (IOM 2009)

Conflict of interest	A set of circumstances that creates a risk that professional judgments or action regarding primary interest will be unduly influenced by a secondary interest
Primary interest	The primary interest that conflict of interest policies seek to protect varies according to the purpose of a professional activity. Primary interests include promoting and protecting the integrity of research, and the welfare of patients. These primary interests are sometimes stated as ends or goals (e.g., pro- moting patient welfare), as obligations (e.g., the physician's obligation to promote patient welfare), or as rights (e.g., the patient's right to have the doctor promote his or her welfare).
Secondary interest	Secondary interests may include not only financial gain but also the desire for professional advancement, recognition for personal achievement, and favors to friends and family or to students and colleagues. The secondary interests are objectionable only when they have greater weight than the primary interest in professional decision making
Conflict	It is a set of circumstances or relationships that create or increase the risk that the primary interests will be neglected as a result of the pursuit of secondary interests. A conflict of interest exists whether or not a particular individual or institution is actually influenced by the secondary interest.
Actual conflict of interest	Arises when secondary interests influence your ability to act with integrity, objectivity, and independence in regards to the assigned task.
Apparent conflict of interest	Arises when secondary interests appear to influence your ability to act with integrity, objectivity, and independence towards the assigned task.
Potential conflict of interest	Arises when you become aware of secondary interests that if acted upon may influence your ability to act with integrity, objectivity, and independence in completing an assigned task.

Determination of conflict of interest

In order to determine if any type of a conflict listed above is possible with your assigned task, ask yourself the following question.

"Would my peers or the general public consider my presentation (judgment) to be biased or would they trust my decision if they knew of this particular situation?"

Disclosure

You are expected to disclose any potential, apparent or actual interests or connections (business, personal, professional, volunteer or other) that appear to influence your ability to act with integrity, objectivity, and independence towards the assigned task.

Over the past 24 months and future 12 months, have you or your spouse received financial support from a commercial entity for any clinical or research activity connected with this task or do you have any other financial relationship with a commercial entity, which might be perceived as having a connection with this task**?**

☐ **NO** - I do not have any relevant financial relationships with any commercial interests. I agree to immediately notify the CAG National Office if a significant financial interest develops at any time prior to or during my participation in the above educational event, presentation or task

☐ **YES** - If yes, please complete the attached table listing identifying which company(ies), what was received and for what role? If the company(ies) is not listed, please provide the additional information in the allotted space below.

Signature: ______________________________ Date: ______________________

FINANCIAL CONFLICT OF INTEREST DISCLOSURE FORM

COMMERCIAL INTEREST	FOR WHAT ROLE?		HOW MUCH?		WHAT WAS RECEIVED?	WHEN?	
Please indicate company number, or specify if needed	Check appropriate answer, or specify if answer not available						
Example: Company A	*Speaker's bureau*	✓	*< 100 US$*		***Honorarium***		
	Consultant		*100 to 499 US$*			*Current*	✓
	Research support		*500 to 999 US$*	✓		*Past < 12 month*	
	Advisory board		*1,000 to 4,999 US$*				
	Employee		*5,000 to 10,000 US$*			*Past 12 to 24 month*	
	Stockholder		*> 10,000 US$*				
	Other:		*Other:*			*Future 12 month*	

List of companies in alphabetical order and identification number

**1**	Akorn	**15**	Johnson and Johnson
**2**	Altaire Pharma	**16**	Lex Pharmaceuticals
**3**	Amend Chemical Company	**17**	LifeCore Biomedical
**4**	American Regent	**18**	Mauna Kea Technology- Cellvizio
**5**	Baker JT	**19**	Medivators
**6**	Boston scientific	**20**	Medsica
**7**	Conmed	**21**	Olympus
**8**	Convidien	**22**	Ovesco
**9**	Cook	**23**	Pentax
**10**	EndoChoice	**24**	Professional Compounding centers
**11**	Endostat	**25**	Seikakagu
**12**	Erbe	**26**	TOP
**13**	Fagron Inc	**27**	Valley Lab
**14**	Fuji, Fujifilm, Fujinon	**28**	US endoscopy

Financial conflict of interest disclosure table

COMMERCIAL INTEREST	FOR WHAT ROLE?		HOW MUCH?		WHAT WAS RECEIVED?	WHEN?	
	Speaker's bureau		< 100 US$				
Company number:	Consultant		100 to 499 US$			Current	
	Research support		500 to 999 US$			Past < 12 month	
______	Advisory board		1,000 to 4,999 US$				
	Employee		5,000 to 10,000 US$			Past 12 to 24 month	
or, specify:	Stockholder		> 10,000 US$				
______________	Other:		Other:			Future 12 month	
	Speaker's bureau		< 100 US$			Current	
Company number:	Consultant		100 to 499 US$				
	Research support		500 to 999 US$			Past < 12 month	
______	Advisory board		1,000 to 4,999 US$				
	Employee		5,000 to 10,000 US$			Past 12 to 24 month	
or, specify:	Stockholder		> 10,000 US$				
______________	Other:		Other:			Future 12 month	
	Speaker's bureau		< 100 US$			Current	
Company number:	Consultant		100 to 499 US$				
	Research support		500 to 999 US$			Past < 12 month	
______	Advisory board		1,000 to 4,999 US$				
	Employee		5,000 to 10,000 US$			Past 12 to 24 month	
or, specify:	Stockholder		> 10,000 US$				
______________	Other:		Other:			Future 12 month	
	Speaker's bureau		< 100 US$			Current	
Company number:	Consultant		100 to 499 US$				
	Research support		500 to 999 US$			Past < 12 month	
______	Advisory board		1,000 to 4,999 US$				
	Employee		5,000 to 10,000 US$			Past 12 to 24 month	
or, specify:	Stockholder		> 10,000 US$				
______________	Other:		Other:			Future 12 month	
	Speaker's bureau		< 100 US$			Current	
Company number:	Consultant		100 to 499 US$				
	Research support		500 to 999 US$			Past < 12 month	
______	Advisory board		1,000 to 4,999 US$				
	Employee		5,000 to 10,000 US$			Past 12 to 24 month	
or, specify:	Stockholder		> 10,000 US$				
______________	Other:		Other:			Future 12 month	
	Speaker's bureau		< 100 US$			Current	
Company number:	Consultant		100 to 499 US$				
	Research support		500 to 999 US$			Past < 12 month	
______	Advisory board		1,000 to 4,999 US$				
	Employee		5,000 to 10,000 US$			Past 12 to 24 month	
or, specify: ______________	Stockholder		> 10,000 US$				
	Other:		Other:			Future 12 month	

Commercial Interest	For what role?		How much?		What was received?	When?	
	Speaker's bureau		< 100 US$			Current	
Company number:	Consultant		100 to 499 US$				
	Research support		500 to 999 US$			Past < 12 month	
______	Advisory board		1,000 to 4,999 US$				
	Employee		5,000 to 10,000 US$			Past 12 to 24 month	
or, specify:	Stockholder		> 10,000 US$				
______________	Other:		Other:			Future 12 month	
	Speaker's bureau		< 100 US$			Current	
Company number:	Consultant		100 to 499 US$				
	Research support		500 to 999 US$			Past < 12 month	
______	Advisory board		1,000 to 4,999 US$				
	Employee		5,000 to 10,000 US$			Past 12 to 24 month	
or, specify:	Stockholder		> 10,000 US$				
______________	Other:		Other:			Future 12 month	
	Speaker's bureau		< 100 US$			Current	
Company number:	Consultant		100 to 499 US$				
	Research support		500 to 999 US$			Past < 12 month	
______	Advisory board		1,000 to 4,999 US$				
	Employee		5,000 to 10,000 US$			Past 12 to 24 month	
or, specify:	Stockholder		> 10,000 US$				
______________	Other:		Other:			Future 12 month	
	Speaker's bureau		< 100 US$			Current	
Company number:	Consultant		100 to 499 US$				
	Research support		500 to 999 US$			Past < 12 month	
______	Advisory board		1,000 to 4,999 US$				
	Employee		5,000 to 10,000 US$			Past 12 to 24 month	
or, specify:	Stockholder		> 10,000 US$				
______________	Other:		Other:			Future 12 month	
	Speaker's bureau		< 100 US$			Current	
Company number:	Consultant		100 to 499 US$				
	Research support		500 to 999 US$			Past < 12 month	
______	Advisory board		1,000 to 4,999 US$				
	Employee		5,000 to 10,000 US$			Past 12 to 24 month	
or, specify:	Stockholder		> 10,000 US$				
______________	Other:		Other:			Future 12 month	
	Speaker's bureau		< 100 US$			Current	
Company number:	Consultant		100 to 499 US$				
	Research support		500 to 999 US$			Past < 12 month	
______	Advisory board		1,000 to 4,999 US$				
	Employee		5,000 to 10,000 US$			Past 12 to 24 month	
or, specify: ______________	Stockholder		> 10,000 US$				
	Other:		Other:			Future 12 month	
	Speaker's bureau		< 100 US$			Current	
Company number:	Consultant		100 to 499 US$				
	Research support		500 to 999 US$			Past < 12 month	
______	Advisory board		1,000 to 4,999 US$				
	Employee		5,000 to 10,000 US$			Past 12 to 24 month	
or, specify:	Stockholder		> 10,000 US$				
______________	Other:		Other:			Future 12 month	

**Commercial Interest**	**For what role?**		**How much?**		**What was received?**	**When?**	
Company number:	Speaker's bureau		< 100 US$			Current	
	Consultant		100 to 499 US$				
______	Research support		500 to 999 US$			Past < 12 month	
	Advisory board		1,000 to 4,999 US$				
or, specify:	Employee		5,000 to 10,000 US$			Past 12 to 24 month	
	Stockholder		> 10,000 US$				
______________	Other:		Other:			Future 12 month	
Company number:	Speaker's bureau		< 100 US$			Current	
	Consultant		100 to 499 US$				
______	Research support		500 to 999 US$			Past < 12 month	
	Advisory board		1,000 to 4,999 US$				
or, specify:	Employee		5,000 to 10,000 US$			Past 12 to 24 month	
	Stockholder		> 10,000 US$				
______________	Other:		Other:			Future 12 month	
Company number:	Speaker's bureau		< 100 US$			Current	
	Consultant		100 to 499 US$				
______	Research support		500 to 999 US$			Past < 12 month	
	Advisory board		1,000 to 4,999 US$				
or, specify:	Employee		5,000 to 10,000 US$			Past 12 to 24 month	
	Stockholder		> 10,000 US$				
______________	Other:		Other:			Future 12 month	
Company number:	Speaker's bureau		< 100 US$			Current	
	Consultant		100 to 499 US$				
______	Research support		500 to 999 US$			Past < 12 month	
	Advisory board		1,000 to 4,999 US$				
or, specify:	Employee		5,000 to 10,000 US$			Past 12 to 24 month	
	Stockholder		> 10,000 US$				
______________	Other:		Other:			Future 12 month	
Company number:	Speaker's bureau		< 100 US$			Current	
	Consultant		100 to 499 US$				
______	Research support		500 to 999 US$			Past < 12 month	
	Advisory board		1,000 to 4,999 US$				
or, specify:	Employee		5,000 to 10,000 US$			Past 12 to 24 month	
	Stockholder		> 10,000 US$				
______________	Other:		Other:			Future 12 month	

Others:

____

____

____

____

____

____

We thank you for taking the time to complete this questionnaire.
