# Supplementary material for: Transparency ethics in practice: Revisiting financial conflicts of interest disclosure forms in clinical practice guidelines
Source: PLoS One. 2017 Aug 25;12(8):e0182856. doi: 10.1371/journal.pone.0182856 (PMC5571907; doi:10.1371/journal.pone.0182856)
Supplement: S3 File — (PDF) [file pone.0182856.s003.pdf]

PARTICIPANT'S PERCEPTIONS ON FINANCIAL CONFLICT OF INTEREST SCENIC CONSENSUS MEETING 2014

Name: ______________________________

Objective

The purpose of this form is to provide personal views and comments on the thresholds for defining relevant financial conflicts of interest in the context of the

Definitions (IOM 2009)

SCENIC Consensus Meeting.

Conflict of interest	A set of circumstances that creates a risk that professional judgments or action regarding primary interest will be unduly influenced by a secondary interest
Primary interest	The primary interest that conflict of interest policies seek to protect varies according to the purpose of a professional activity. Primary interests include promoting and protecting the integrity of research, the welfare of patients, and the quality of medical education. These primary interests are sometimes stated as ends or goals (e.g., pro- moting patient welfare), as obligations (e.g., the physician's obligation to promote patient welfare), or as rights (e.g., the patient's right to have the doctor promote his or her welfare).
Secondary interest	Secondary interests may include not only financial gain but also the desire for professional advancement, recognition for personal achievement, and favors to friends and family or to students and colleagues. The secondary interests are objectionable only when they have greater weight than the primary interest in professional decision making
Conflict	It is a set of circumstances or relationships that create or increase the risk that the primary interests will be neglected as a result of the pursuit of secondary interests. A conflict of interest exists whether or not a particular individual or institution is actually influenced by the secondary interest.
Actual conflict of interest	Arises when secondary interests influence your ability to act with integrity, objectivity, and independence in regards to the assigned task.
Apparent conflict of interest	Arises when secondary interests appear to influence your ability to act with integrity, objectivity, and independence towards the assigned task.
Potential conflict of interest	Arises when you become aware of secondary interests that if acted upon may influence your ability to act with integrity, objectivity, and independence in completing an assigned task.

Determination of financial conflicts of interest

1. Do you believe that participants should disclose actual, potential or perceived financial conflicts of interest for this meeting, and why?

2. In your opinion, what is the minimal amount that needs to be disclosed?

3. In your opinion, what is the time frame (in months) that should be included when disclosing financial conflicts of interests?

4. In your opinion, is the disclosure of financial conflicts of interest sufficient to allow full participation in the consensus process of the guideline development?

If not, what measures should be taken to manage the participation of participants with such financial conflicts of interest?

5. Do you have any additional comments, concerns or suggestions regarding the disclosure and management of financial conflicts of interest of this consensus guideline?

6. Are you aware of any national or institutional financial conflicts of interest policies in your practicing country that addresses financial conflicts of interest and/or disclosure thresholds?

We thank you for taking the time to complete this questionnaire.

Participant's demographic information

Country: ____

Affiliated institution: ___________________________________

Age: ______

Years in practice: _________

Field of expertise pertaining to your SCENIC participation:
(Please select most appropriate)

___ Clinician (Gastroenterology) ___ Clinician (Surgery) ___ Methodologist
___ Nurse ___ Patient

Signature: ___________________________ Date: ___________________
