# Supplementary material for: Transparency ethics in practice: Revisiting financial conflicts of interest disclosure forms in clinical practice guidelines
Source: PLoS One. 2017 Aug 25;12(8):e0182856. doi: 10.1371/journal.pone.0182856 (PMC5571907; doi:10.1371/journal.pone.0182856)
Supplement: S4 File — (DOCX) [file pone.0182856.s004.docx]

**COMPETING INTERESTS**

**Authors’ Competing Interests**

The authors of this manuscript (YL DJJ NS TK LL KM RS VS AB) have no competing interests to disclose.

**Financial conflict of interest disclosures from the SCENIC clinical practice guideline’s participants [1]**

The following is a summary of the financial disclosure provided by the following participants: Alan Barkun (AB), James E. East (JE), Francis A. Farraye (FF), Brian Feagan (BF), John Ioannidis (JI), Tonya Kaltenbach (TK), Ralf Kiesslich (RK), Michael Krier (MK), Loren Laine (LL), Takayuki Matsumoto (TM), Robert P. McCabe (RM), Ken- neth R. McQuaid (KMc), Fabrizio Michelassi (FM), Klaus Mönkemüller (KM), Robert Odze (RO), Michael Picco (MP), David T. Rubin (DR), Michele Rubin (MR), Carlos A. Rubio (CR), Matt D. Rutter (MRu), Andres Sanchez-Yague (AS-Y), Silvia Sanduleanu (SS), Amandeep Shergill (ASh), Roy Soe- tikno (RS), Venkataraman Subramanian (VS), Thomas Ull- man (TU), Fernando Velayos (FV), Douglas Yakich (DY), Yu- Xiao Yang (Y-XY).

No significant financial interest to report:

JI, RK, MK, LL, TM, RM, KMc, FM, RO, MP, MR, CR, AS-Y, SS, ASh, VS, FV, DY, Y-XY.

Yes, a significant financial interest as follows:

Advisory Board: Abbvie (JE, BF), Amgen (BF), Astra- Zeneca (BF), Avaxia Biologics (BF), Braintree (FF), Bristol Myers Squibb (BF), Celgene (BF), Centocor (BF), Cosmo Pharmaceuticals (JE), Elan/Biogen (BF), Entera Health (FF), Ferring (BF), Genentech (TU), Janssen (FF, BF), Merck (BF), Novartis (BF), NovoNordisk (BF), Olympus (AB), Pendopharm (AB), Pfizer (BF), Prometheus (BF), Salix Pharmaceuticals (FF, BF), Takeda (BF), Teva Pharmaceu- ticals (BF), Tillotts Pharmaceuticals (BF), UCB Pharma- ceuticals (BF).

Honorarium: Abbvie (JE, BF), Actogenix (BF), Albireo Pharmaceuticals (BF), Amgen (BF), AstraZeneca (AB, BF), Avaxia Biologics (BF), Axcan (BF), Baxter (BF), Boehringer-Ingelheim (BF), Boston Scientific (AB), Bristol Myers Squibb (BF), Calypso Biotech (BF), CDX (TU), Cel- gene (BF), Centocor (BF), Cook (AB, KM), Cosmo Phar- maceuticals (JE), Elan/Biogen (BF),EnGene (BF), Ferring (BF), Genentech (TU), GiCare Pharmaceuticals (BF), Gilead (BF), Given Imaging (BF), GSK (BF), Ironwood Pharma- ceuticals (BF), Janssen Biotech (Centocor) (BF), Janssen (TU, BF), Kyowa Kakko Kirin (BF), Lexicon (BF), Lilly (BF), Merck (BF), Millennium (BF), Nektar (BF), Novartis (BF), NovoNordisk (BF), Olympus, (AB, MRu), Ovesco (KM), Pendopharm (AB), Prometheus Laboratories (BF), Pro- metheus Therapeutics and Diagnostics (BF), Pfizer (BF, TU), Receptos (BF), Roche/Genentech (BF), Salix Phar- maceuticals (BF), Serono (BF), Shire (BF), Sigmoid Phar- maceuticals (BF), Synergy Pharmaceuticals (BF), Takeda (AB, BF), Teva Pharmaceuticals (BF), Tillotts Pharmaceu- ticals (BF), UCB Pharmaceuticals (BF), Vertex Pharma- ceuticals (BF), Warner-Chilcott (BF), Wyeth (BF), Zealand (BF), Zyngenia (BF).

Research Support: Abbott (BF), Abbvie (BF, DR), Amgen (BF), AstraZeneca (BF), Boston Scientific (AB), Bristol Myers Squibb (BF), Cook (AB), Cosmo Pharmaceuticals (JE), Cubist (FF), Elan Pharmaceuticals (DR), Genentech (BF, TU), Jans- sen Biotech (Centocor) (BF), Janssen (BF), Millennium (BF), Olympus (JE, RS, TK), Pfizer (BF), Prometheus Pharmaceu- ticals (FF, DR), Receptos (BF), Santarus (BF), Sanofi (BF), Shire (DR), Tillotts (BF), UCB Pharmaceuticals (BF), Warner Chilcott (DR).

Speaker’s Bureau: Abbvie (JE, BF), AstraZeneca (AB), Cook (AB, KM), Cosmo Pharmaceuticals (JE), Janssen (BF, TU), Ovesco (KM), Olympus (RS, MRu), Pendopharm (AB), Takeda (AB, BF), UCB Pharmaceuticals (BF), Warner- Chilcott (BF).

Consultant: Abbvie (BF, DR), Actogenix (BF), Albireo Pharmaceuticals (BF), Amgen (BF), AstraZeneca (BF), Avaxia Biologics (BF), Axcan (BF), Baxter (BF), Boehringer- Ingelheim (BF), Bristol Myers Squibb (BF, DR), Calypso Biotech (BF), CDX (TU), Celgene (BF), Cook (AB), Elan/ Biogen (BF, DR), EnGene (BF), Emmi (DR), Ferring (BF), Genentech (BF, TU), GiCare Pharmaceuticals (BF), Gilead (BF), Given Imaging (BF, DR), GSK (BF), Ironwood Phar- maceuticals (BF, DR), Janssen Biotech (Centocor) (FF, BF, DR, TU), Janssen (BF), Kyowa Kakko Kirin (BF), Lexicon (BF), Lifecore Biomedical (DR), Lilly (BF), Merck (BF), Nektar (BF), NovoNordisk (BF), Olympus (TK), Olympus (RS), Pfizer (BF, TU), Prometheus Pharmaceuticals (DR), Prometheus Therapeutics and Diagnostics (BF), Receptos (BF), Roche/Genentech (BF), Salix Pharmaceuticals (BF), Santarus (FF, DR), Serono (BF), Shire (BF), Sigmoid Phar- maceuticals (BF), Synergy Pharmaceuticals (BF), Takeda (BF), Teva Pharmaceuticals (BF), Tillotts (BF), Takeda- Millennium (BF, DR), Telsar Pharmaceuticals (DR), UCB Pharmaceuticals (BF, DR), Vertex Pharmaceuticals (BF, DR), Warner-Chilcott (BF), Wyeth (BF), Zealand (BF), Zyngenia (BF).

**References**

1. Laine L, Kaltenbach T, Barkun A, McQuaid KR, Subramanian V, Soetikno R, et al. SCENIC international consensus statement on surveillance and management of dysplasia in inflammatory bowel disease. Gastroenterology. 2015;148(3):639-51 e28.
